# Supplementary material for: Structural insights into POT1-TPP1 interaction and POT1 C-terminal mutations in human cancer
Source: Nat Commun. 2017 Apr 10;8:14929. doi: 10.1038/ncomms14929 (PMC5394241; doi:10.1038/ncomms14929)
Supplement: Supplementary Information — Supplementary Figures and Supplementary Tables [file ncomms14929-s1.pdf]

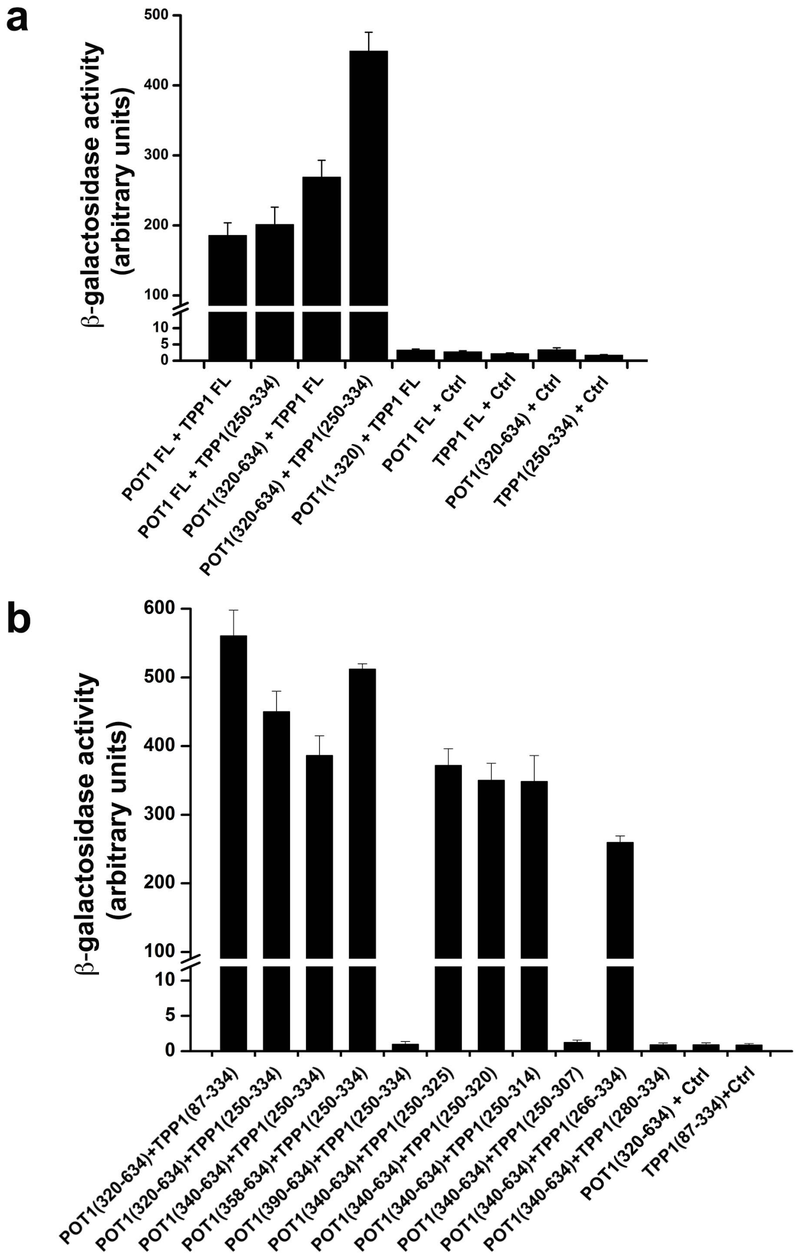

Supplementary Figure 1. Yeast two-hybrid analysis of the POT1-TPP1 interaction. Interaction between LexA-POT1 and GAD-TPP1 was analyzed by measuring the β-galactosidase activity produced by the reporter gene. Error bar (s.d.) was derived from three independent β-galactosidase measurements. **(a)** Interaction between full-length TPP1 with different fragments POT1. **(b)** Interactions between POT1C (residues 320-634) and different fragments of TPP1.

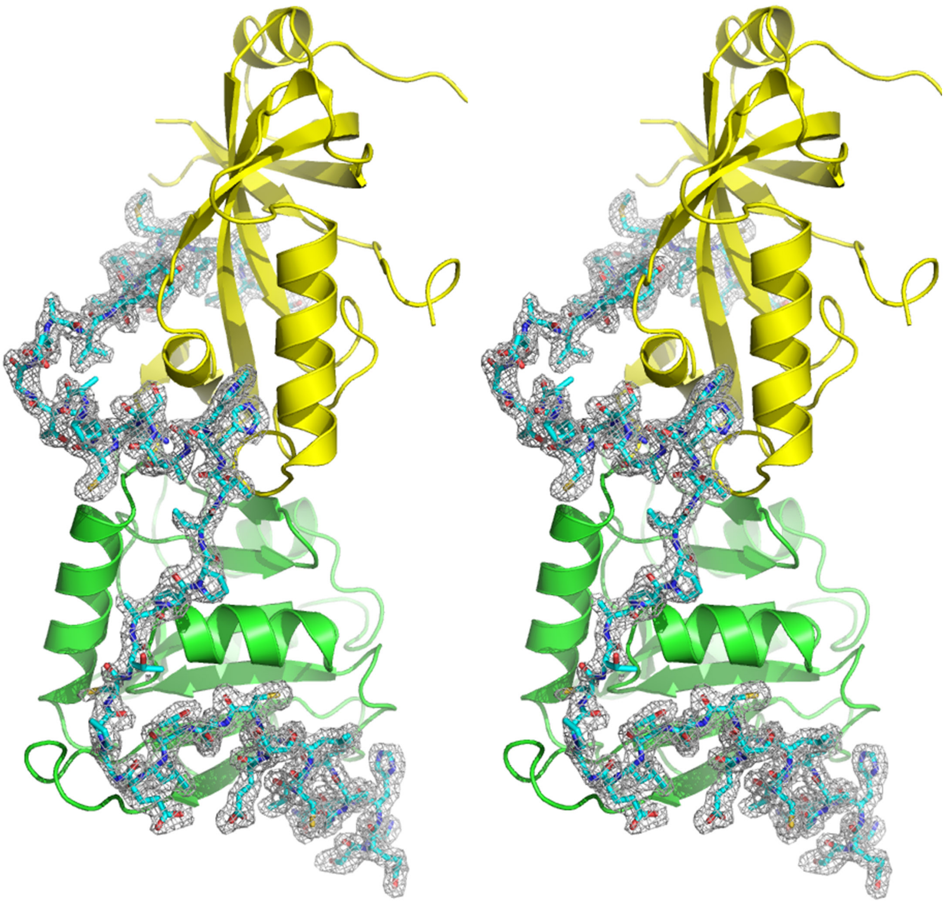

Supplementary Figure 2. Stereo view of the overall structure of the POT1C-$TPP1_{PBM}$ complex. The electron density (2Fo-Fc) map of $TPP1_{PBM}$, contoured at 1.0 σ, is shown in the complex.

a

*Hs*POT1$_{HJRL}$ and *Af*Hjc

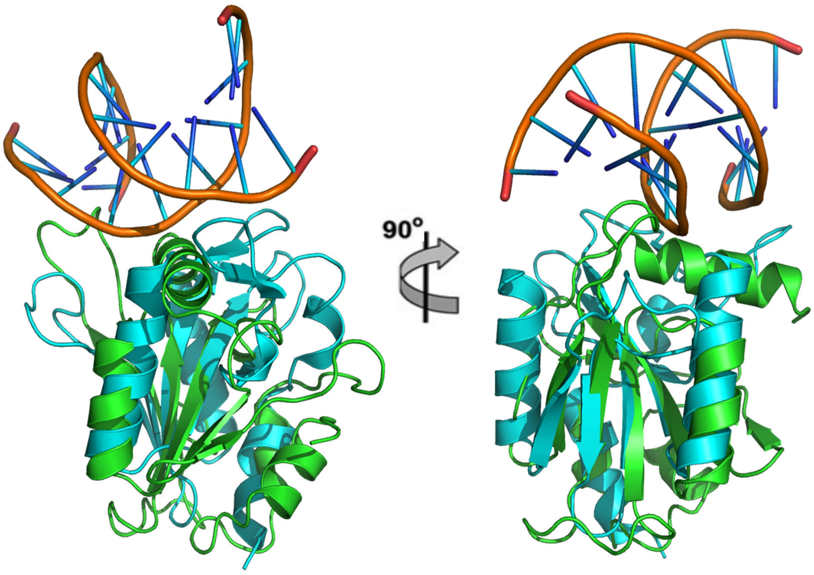

rmsd: 3.3 Å, N_{Res}: 80

b

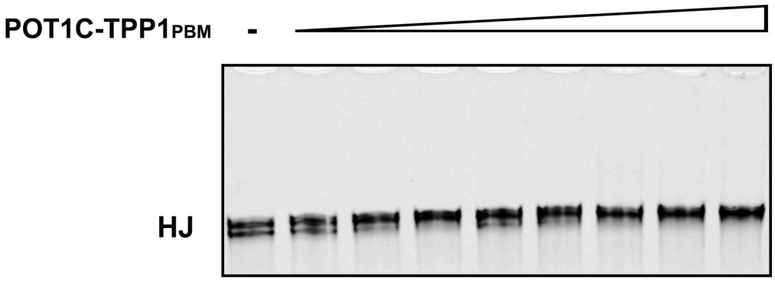

Supplementary Figure 3. Structural and functional studies of POT1C. (a) Superposition of *Hs*POT1$_{HJRL}$ on the structure of the *Af*Hjc. Human POT1$_{HJRL}$ is colored in green and *Archaeoglobus fulgidus* Holliday Junction resolvase Hjc in cyan. The rmsd value and the number of residues used for superposition are listed. **(b)** Electrophoretic mobility shift assay (EMSA) showed that the POT1C-TPP1$_{PBM}$ cannot bind Holliday junction. The binding mixture contained 20 nM Holliday junction substrate and 0 to 64 μM of purified POT1C-TPP1$_{PBM}$ complex.

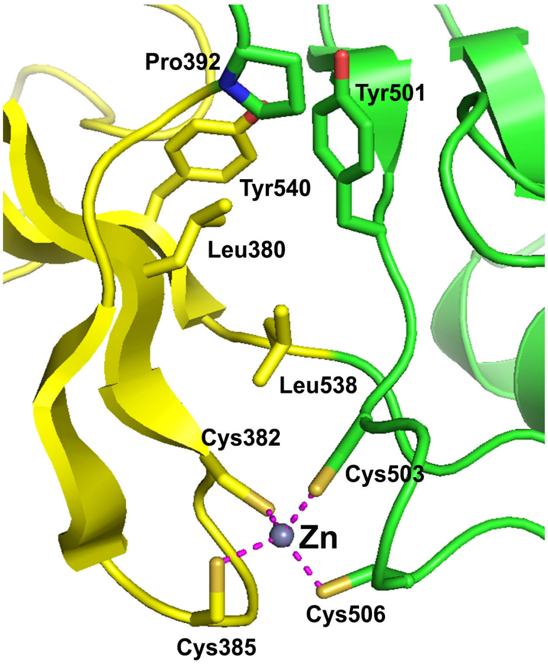

Supplementary Figure 4. The interface between the OB fold and the HJRL domain of POT1. POT1$_{OB3}$ is colored in yellow and POT1$_{HJRL}$ is colored in green. Residues at the domain interface are represented in stick model.

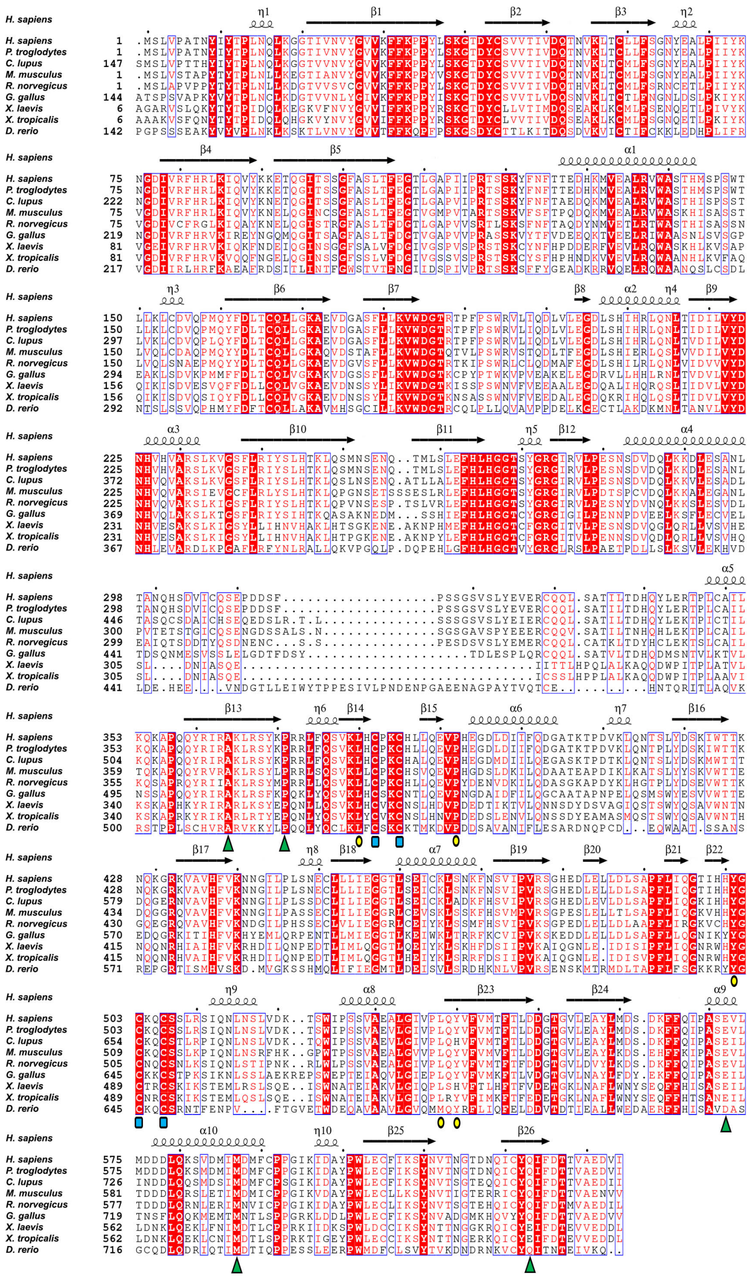

Supplementary Figure 5. Multiple sequence alignment of vertebrate POT1 homologues. POT1 is highly conserved from human to zebrafish. The secondary structures (α, α-helix; β, β-strand; η, 3_{10}-helix) of human POT1 are labeled on the top of each bars. *Homo sapiens*, NP_056265.2; *Pan troglodytes*, XP_009452413.1; *Canis lupus*, XP_005628497.1; *Mus musculus*, NP_598692.1; *Rattus norvegicus*, NP_001019493.1; *Gallus gallus*, NP_996875.1; *Xenopus laevis*, NP_001084422.1; *Xenopus tropicalis*, NP_998876.1; *Danio rerio*, NP_001232889.1. Green triangles show residues involve in C-terminal mutations in human diseases. Blue rectangles stand for cysteines that chelate the Zinc ion. Yellow dots represent the most credible residues that found in XL-MS.

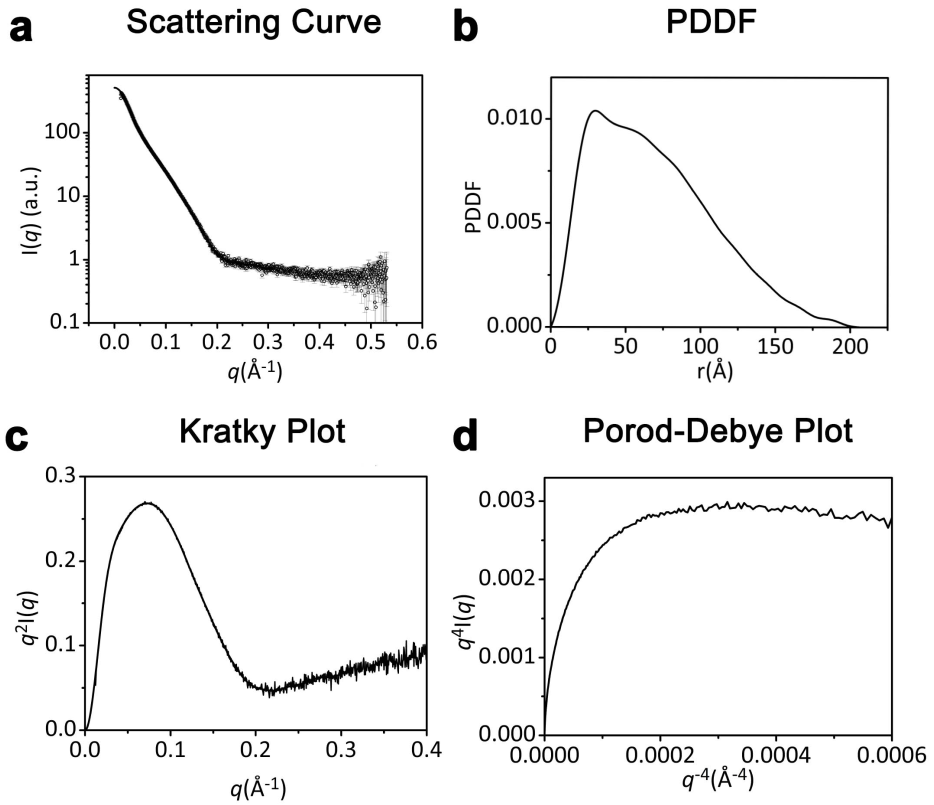

Supplementary Figure 6. SAXS analysis of the POT1-TPP1N-T10 complex. (a) Scattering intensity in arbitrary units versus momentum transfer q in $Å^{-1}$. **(b)** Pair distance distribution function (PDDF). **(c)** Kratky plot. **(d)** Porod-Debye plot.

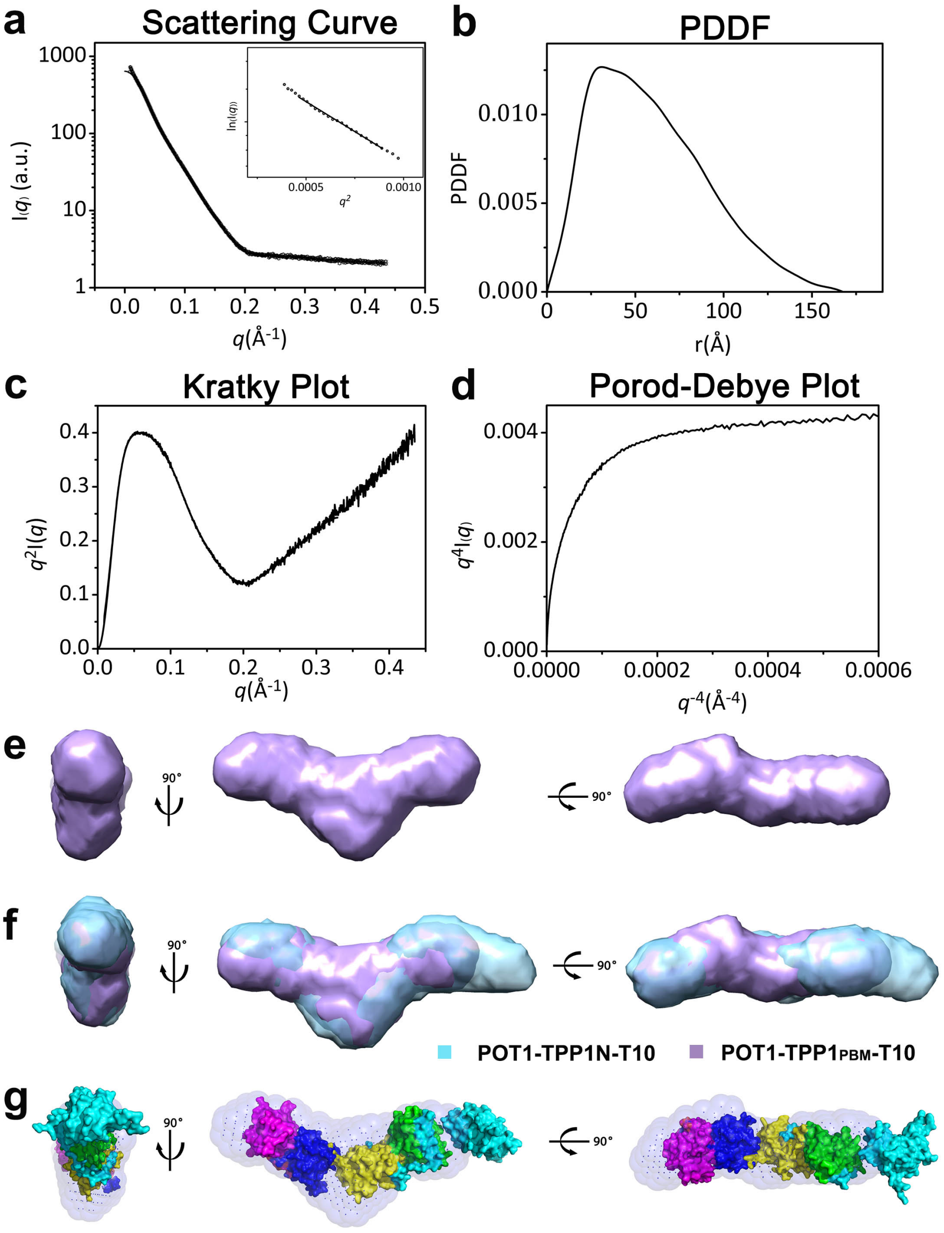

Supplementary Figure 7. SAXS analysis of the POT1-TPP1$_{PBM}$-T10 complex. (a) Scattering intensity in arbitrary units versus momentum transfer q in Å^{-1}. The inset in (a) shows the Guinier region of the scattering curve with a linear fit line. **(b)** Pair distance distribution function (PDDF). **(c)** Kratky plot. **(d)** Porod-Debye plot. **(e)** Three views of the symmetric V-shaped envelop of the POT1-TPP1$_{PBM}$-T10 complex. The envelope is colored in purple. **(f)** Superposition of the envelope of the POT1-TPP1$_{PBM}$-T10 and the POT1-TPP1N-T10 complexes, colored in purple and light blue, respectively. The extra density on the POT1-TPP1N-T10 envelope indicates the position of TPP1$_{OB}$. **(g)** Docking of the crystal structures of human TPP1$_{OB}$, POT1$_{OB3\text{-}HJRL}$-TPP1$_{PBM}$ and POT1$_{OB1\text{-}OB2}$-ssDNA into the envelope of POT1-TPP1$_{PBM}$-T10 complex. POT1 OB1, OB2, OB3, and HJRL, and TPP1 are colored in magenta, blue, yellow, green, and cyan, respectively. Envelope of the POT1-TPP1$_{PBM}$-T10 complex is colored in purple.

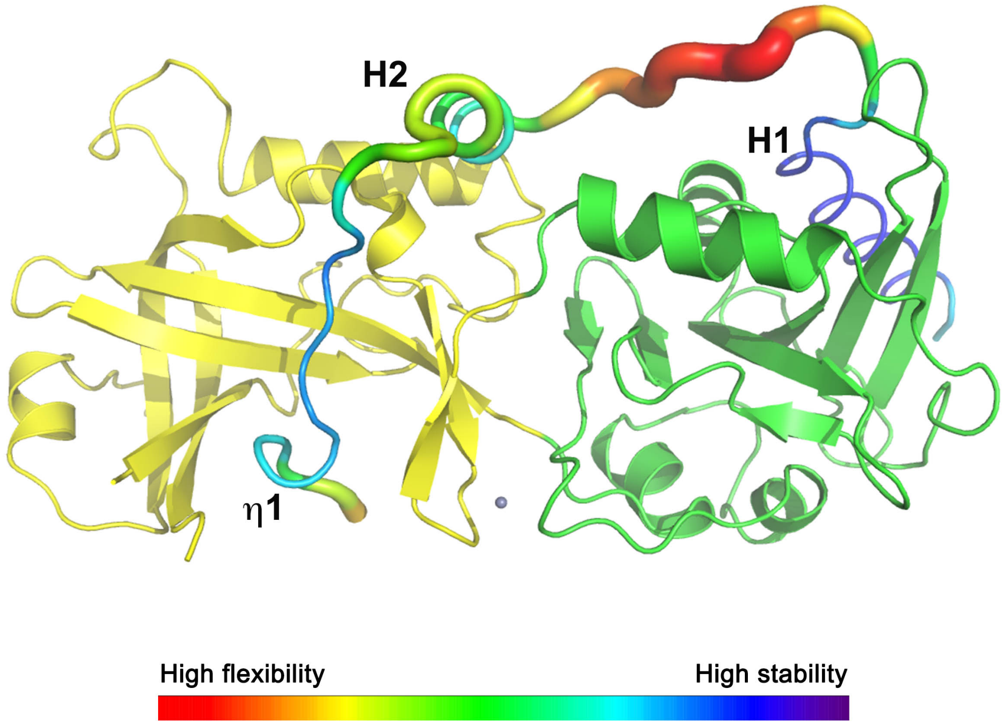

Supplementary Figure 8. The loop region between helices H1 and H2 of TPP1$_{PBM}$ is highly flexible. TPP1$_{PBM}$ is presented as B factor putty mode and the loop linking helices H1 and H2 is high flexible and make no direct contact with POT1C.

Number	WT and mutant POT1
1	WT
2	W424E+F438R (Module 1)
3	Q580R+D584R (Module 2)
4	Q623H (Module 3)
5	W424E+F438R+Q580R+D584R (Module 1+2)
6	W424E+F438R+Q623H (Module 1+3)
7	Q580R+D584R+Q623H (Module 2+3)
8	Vector

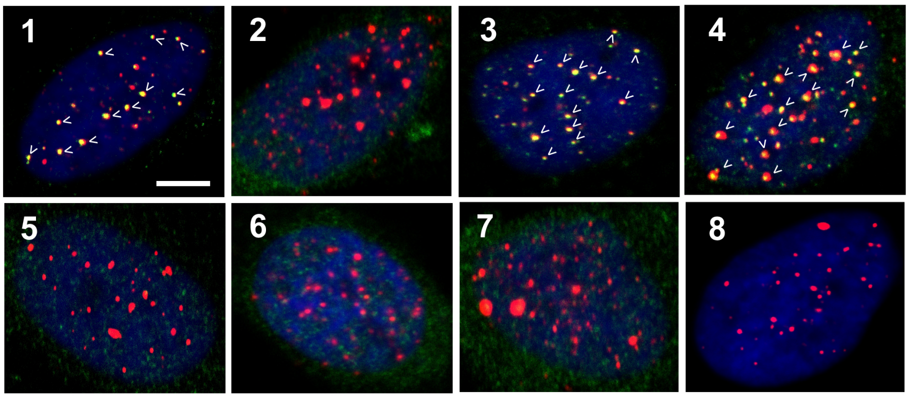

Supplementary Figure 9. Telomere localization analysis of mutant POT1 proteins in U2OS cells. Cells were immunostained with anti-Flag (green) and hybridized with Cy5-$(CCCTAA)_4$ probe (red) to detect telomere location of WT and mutant POT1 proteins. Nuclei were stained with DAPI (blue). Arrows point to Flag-POT1 foci at telomeres. Scale bar is 5 microns in length.

a

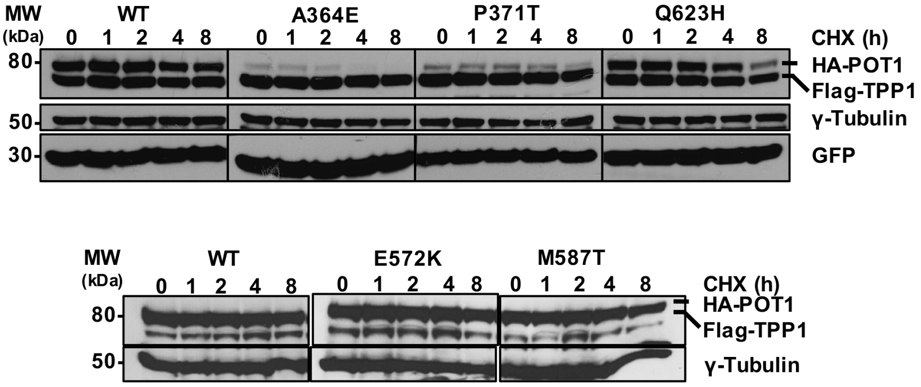

b

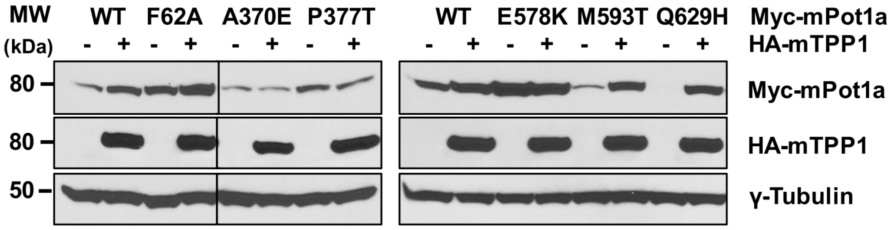

Supplemental figure 10. Stability analyses of WT and mutant human and mouse POT1 proteins. (a) Stability of WT and mutant POT1 in the presence of cycloheximide. (top) WT HA-POT1, HA-POT1^{A364E}, HA-POT1^{P371T}, and HA-POT1^{Q623H} cloned in MSCV-IRES-GFP vectors and co-transfected with Flag-hTPP1 (ratio of 3:1 POT1:TPP1) into 293T cells. 30μg/ml of CHX was added 48hr after transfection. At the indicated time points, cells were harvested, lysed and the expression of HA-POT1 and Flag-TPP1 was analyzed by western blot. γ-tubulin was used for total loading controls and anti-GFP was used to show unaltered expression of GFP in the presence of CHX. (bottom) The HA-POT1^{E572K} and HA-POT1^{M587T} mutants were cloned in MSCV-IRES-Puro vector. Transfection and detection procedures were identical as described above. **(b)** Characterization of mPOT1a C-terminal mutants. Co-expression of WT and mutant mPOT1a with or without mTPP1 in 293T cells. γ-tubulin served as a loading control.

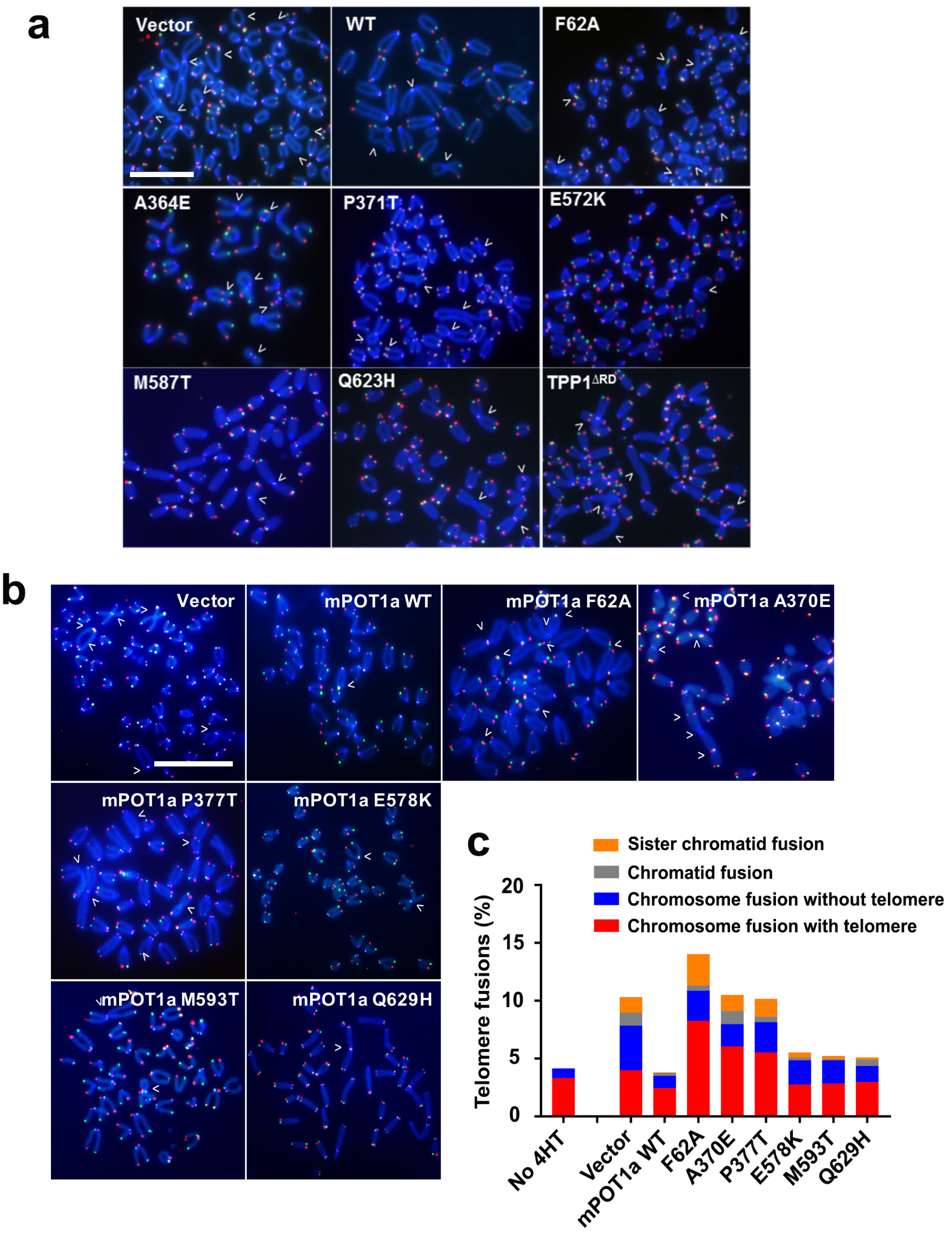

Supplementary Figure 11. POT1C OB3 mutants cannot protect telomeres from engaging in a DDR. (a) *CAG-Cre*^{ER}; *mPOT1a*$^{F/F}$; *mPOT1b*$^{-/-}$ MEFs reconstituted with WT POT1 or the indicated POT1 mutants were treated with 4-HT, metaphase spreads prepared and analyzed by PNA-FISH. Arrowheads point to representative telomere fusions. Scale bar is 25 microns in length. **(b)** *CAG-Cre*^{ER}; *mPOT1a*$^{F/F}$; *mPOT1b*$^{-/-}$ MEFs reconstituted with WT mPOT1a or the indicated mPOT1a mutants were treated as described above. Arrowheads point to representative telomere fusions. Scale bar is 25 microns in length. **(c)** Quantitation of the percentage of telomere fusions in *CAG-Cre*^{ER}; *mPOT1a*$^{F/F}$; *mPOT1b*$^{-/-}$ MEFs reconstituted with the indicated mPOT1a constructs and then treated with 4-HT. A minimum of 45 metaphases were scored per genotype.

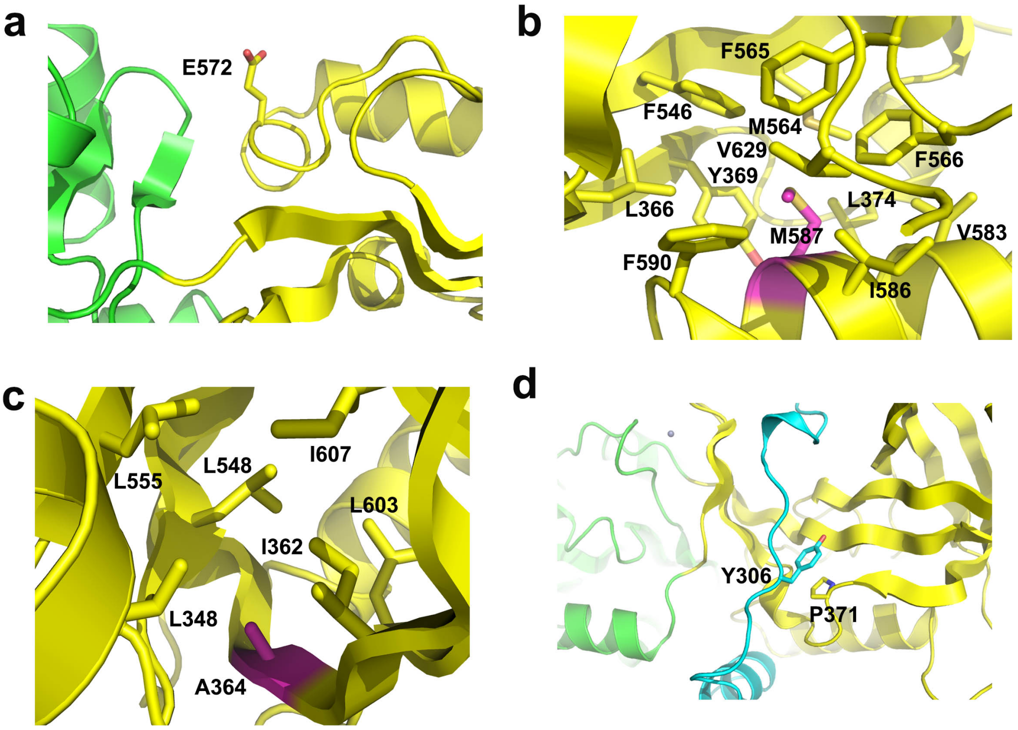

Supplementary Figure 12. Structural implication of POT1 C-terminal mutations. (a-d) Ribbon diagrams show the structural environment of POT1 residues E572 (a), M587 (b), A364 (c), and P371 (d)) that are mutated in human cancers.

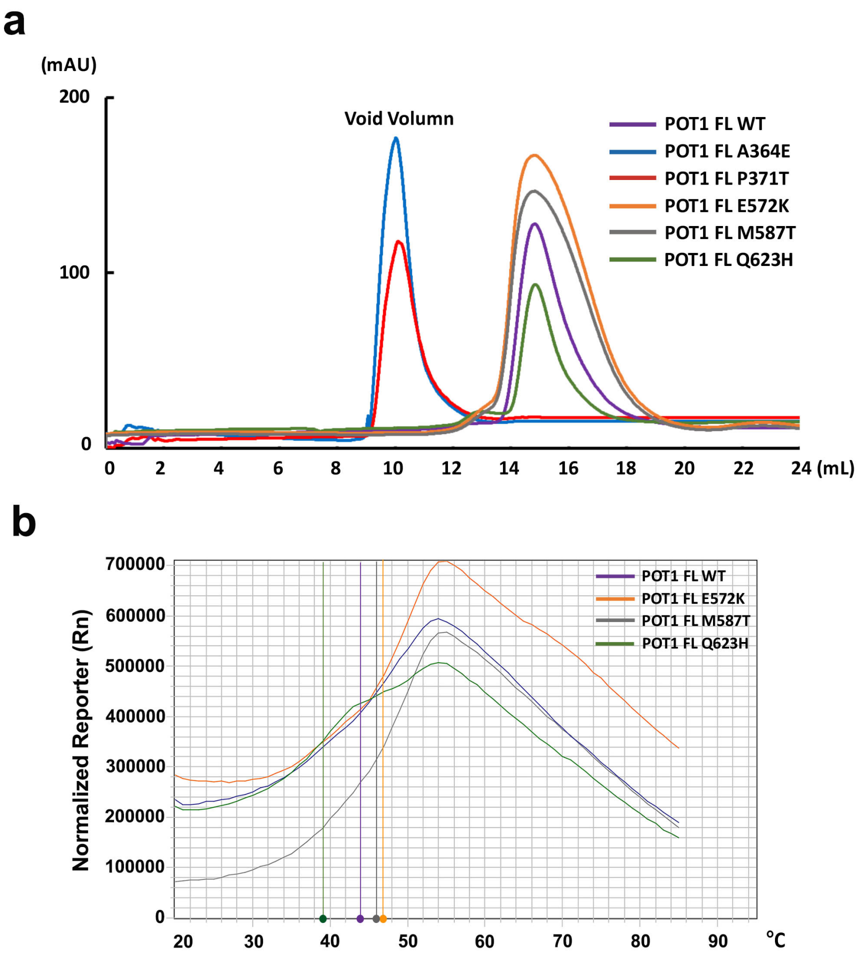

Protein	Tm (°C)
POT1 FL WT	44
POT1 FL E572K	47
POT1 FL M587T	46
POT1 FL Q623H	39

Supplementary Figure 13. Stability analysis of mutant POT1 protein found in cancers. (a) Gel filtration profile of Superdex 200 shows that purified POT1 FL A364E and P371T are aggregates, while POT1 FL WT, E572K, M587T and Q623H show good profile relatively. **(b)** Melting curve shows that POT1 Q623H is unstable relative to POT1 WT, E572K and M587T.

Supplementary Table 1. Crystal data collection and refinement statistics

	POT1C-TPP1$_{PBM}$ (Native)	POT1C-TPP1$_{PBM}$ (SeMet-SAD)
**Data collection**		
Wavelength	0.97876	0.97876
Space group	$P2_12_12_1$	$P2_12_12_1$
Cell dimensions		
a, b, c (Å)	49.23, 87.82, 87.12	51.02, 86.57, 89.34
α, β, γ (°)	90.00, 90.00, 90.00	90.00, 90.00, 90.00
Resolution (Å)	2.1	3.1
R_{merge}	0.129 (0.619) *	0.142 (0.491) *
$I / \sigma I$	13.3 (2.6) *	11.1 (2.8) *
Completeness (%)	98.1 (93.6) *	91.4 (93.3) *
Redundancy	6.2 (5.2) *	5.6 (5.8) *
**Refinement**		
Resolution (Å)	43.91-2.10	
No. of reflections	21,417	
R_{work} / R_{free} (%)	19.6/24.8	
No. of atoms		
POT1C	2,299	
TPP1$_{PBM}$	375	
Zinc	1	
Water	140	
B-factors		
POT1C	28.8	
TPP1$_{PBM}$	41.8	
Zinc	31.7	
Water	31.1	
R.m.s. deviations		
Bond lengths (Å)	0.006	
Bond angles (°)	1.138	
Ramanchandran plot		
Favored region	99.1%	
Allowed region	100.0%	
Outlier region	0.0%	

*Highest resolution shell is shown in parenthesis

Supplementary Table 2. Molecular parameters of POT1-TPP1N-T10 analyzed by SAXS

Concentration (mg/mL)	R_g (nm)	D_{max} (nm)
10	5.77 ± 0.05	20.5
5	5.73 ± 0.03	20.6
2.5	5.50 ± 0.07	19.6

Supplementary Table 3. Molecular parameters of POT1-TPP1$_{PBM}$-T10 analyzed by SAXS

Concentration (mg/mL)	R_g (nm)	D_{max} (nm)
10	5.05 ± 0.03	16.8
5	5.03 ± 0.05	16.7
1	5.03 ± 0.03	16.7
